# Socio-economic difference in purchases of ultra-processed foods in Australia: an analysis of a nationally representative household grocery purchasing panel

**DOI:** 10.1186/s12966-022-01389-8

**Published:** 2022-12-12

**Authors:** Daisy H. Coyle, Liping Huang, Maria Shahid, Allison Gaines, Gian Luca Di Tanna, Jimmy Chun Yu Louie, Xiongfei Pan, Matti Marklund, Bruce Neal, Jason H. Y. Wu

**Affiliations:** 1 Faculty of Medicine, The George Institute for Global Health, University of New South Wales, Sydney, NSW 2042 Australia; 2 Department of Epidemiology and Biostatistics, Faculty of Medicine, School of Public Health, Imperial College London, London, SW7 2AZ UK; 3 School of Biological Science, Faculty of Science, The University of Hong Kong, Hong Kong, 999077 China; 4 Key Laboratory of Birth Defects and Related Diseases of Women and Children (Sichuan University), Ministry of Education, West China Second University Hospital, Sichuan University, Chengdu, 610041 Sichuan China

**Keywords:** Ultra-processed food, Socio-economic status, Diet, Nutrition, Food supply, Processing

## Abstract

**Background:**

Consumption of ultra-processed foods is associated with increased risk of obesity and non-communicable diseases. Little is known about current patterns of ultra-processed foods intake in Australia. The aim of this study was to examine the amount and type of ultra-processed foods purchased by Australian households in 2019 and determine whether purchases differed by socio-economic status (SES). We also assessed whether purchases of ultra-processed foods changed between 2015 and 2019.

**Methods:**

We used grocery purchase data from a nationally representative consumer panel in Australia to assess packaged and unpackaged grocery purchases that were brought home between 2015 to 2019. Ultra-processed foods were identified according to the NOVA system, which classifies foods according to the nature, extent and purpose of industrial food processing. Purchases of ultra-processed foods were calculated per capita, using two outcomes: grams/day and percent of total energy. The top food categories contributing to purchases of ultra-processed foods in 2019 were identified, and differences in ultra-processed food purchases by SES (Index of Relative Social Advantage and Disadvantage) were assessed using survey-weighted linear regression. Changes in purchases of ultra-processed foods between 2015 to 2019 were examined overall and by SES using mixed linear models.

**Results:**

In 2019, the mean ± SD total grocery purchases made by Australian households was 881.1 ± 511.9 g/d per capita. Of this, 424.2 ± 319.0 g/d per capita was attributable to purchases of ultra-processed foods, which represented 56.4% of total energy purchased. The largest food categories contributing to total energy purchased included mass-produced, packaged breads (8.2% of total energy purchased), chocolate and sweets (5.7%), biscuits and crackers (5.7%) and ice-cream and edible ices (4.3%). In 2019, purchases of ultra-processed foods were significantly higher for the lowest SES households compared to all other SES quintiles (*P* < 0.001). There were no major changes in purchases of ultra-processed foods overall or by SES over the five-year period.

**Conclusions:**

Between 2015 and 2019, ultra-processed foods have consistently made up the majority of groceries purchased by Australians, particularly for the lowest SES households. Policies that reduce ultra-processed food consumption may reduce diet-related health inequalities.

**Supplementary Information:**

The online version contains supplementary material available at 10.1186/s12966-022-01389-8.

## Introduction

The global food supply has seen major shifts in recent years marked by a rapid increase in the production and consumption of mass-produced, heavily marketed, ultra-processed foods [1, 2]. These are industrially manufactured, ready-to-eat or heat foods that bear little resemblance to the ingredients from which they originally derived [3]. Examples of ultra-processed foods include some breakfast cereals, confectionary, reconstituted meat products and sugar-sweetened beverages. Ultra-processed foods are highly palatable, cheap, convenient, and in many countries have displaced traditional diets based on unprocessed and minimally processed foods [1, 4].

There are growing concerns regarding the adverse health consequences of consuming ultra-processed foods [5, 6]. High intakes of ultra-processed foods have been associated with low overall diet quality [7–10], which appears to be largely driven by the fact these products tend to be high in unfavourable nutrients including added sugar, sodium, and trans fatty acids [8, 11, 12]. In large population-based studies, higher intake of ultra-processed foods has been associated with increased risk of weight gain, diabetes, cardiovascular disease, depression, cancer, and all-cause mortality [5, 13, 14]. There is also emerging evidence from clinical trials on the causal effect of ultra-processed foods on weight gain, possibly through the impact on appetite hormones and disruptions to gut-brain signalling [15, 16]. The emerging health risks of ultra-processed foods is concerning, particularly for socio-economically disadvantaged individuals who already experience a disproportionate burden of diet-related disease due to a lack of affordability and accessibility to high-quality fresh produce such as fruits and vegetables [17–21].

Given the mounting evidence demonstrating that the level of processing a food has undergone relates to overall diet quality and development of non-communicable diseases (NCDs), there is a need for data relating to the amount and type of ultra-processed foods purchased by consumers, especially given the diverse and fast-changing nature of the food supply [2, 22]. Such data may help to guide consumer education and policies aimed at improving the food environment. It is within this context that the primary aims of this study were to examine the amount and types of ultra-processed foods purchased by Australian households in 2019, and to investigate whether household purchases of ultra-processed foods differed according to socio-economic status (SES). We assessed also changes in purchases of ultra-processed foods by SES between 2015 and 2019.

## Material and methods

This study was approved by The University of New South Wales Human Research Ethics Committee (approval number HC200244).

### Study design and population

This study used household purchase data from the NielsenIQ Homescan Consumer Panel, a dataset that contains household-level food and beverage purchase data from a panel of approximately 10,000 Australian households. These households are recruited to be broadly representative of the demographic composition and geographic location of Australian households [23, 24]. Participating households are provided with a handheld electronic scanner and are asked to scan the barcode of all foods and beverages brought into the home from all retail outlets including supermarkets, grocers, and convenience stores. Data on non-barcoded items such as deli meats, fresh bakery items and fresh unpackaged fruits and vegetables are also collected through use of a scanning guide booklet provided to the households by NielsenIQ [23, 24]. No data are collected on food purchased and consumed outside of the home, such as take-away and restaurant foods. For the present analyses, both barcoded and non-barcoded items were included.

Sociodemographic information is also collected from households, including information about ethnicity, education level of the main shopper in the household, household income, lifestage (e.g., adult households and young families), and age and sex of all household members. To capture annual changes in purchasing behaviours from 2015 to 2019, all household purchases were aggregated for each calendar year over the five-year period (January 1^st^ – December 31^st^).

### Household eligibility

To exclude households with potentially unreliable data, we applied criteria set by NielsenIQ. We removed households who: (i) were not on the panel for the entire 52-week time frame; (ii) did not report purchase data (at least one barcode per week) for at least 50% of the weeks within a 12-month timeframe; (iii) were missing any demographic information; (iv) did not meet the minimum spend criteria (≥ $5 on average for each week for all purchases). As previously described, to further reduce the potential impact of under-reporting, we excluded households with the lowest annual food and beverage expenditure (< 2.5^th^ percentile defined separately for single-member households and multi-member households) [23–27].

### Nutrition information

To determine the energy content of each food and beverage product at the time of purchase, barcoded products in the Homescan dataset were linked with corresponding nutrition information from the FoodSwitch nutrition composition database [28]. This database contains nutrition information for more than 80,000 packaged foods and beverages that have been available for sale in Australia since January 2013. Most of the data (~ 60% of all products) are captured by trained data collectors through in-store surveys at five large Australian supermarkets owned by Aldi, Coles, Harris Farm, Independent Grocers of Australia (IGA) and Woolworths in the Sydney metropolitan area [29]. Images of the pack of each food and beverage product are captured (front of pack, nutrient declaration, ingredients list, manufacturer details), using a bespoke smartphone application. The product name, brand name, package size (g) and nutrient content per 100 g/mL and per serve are then extracted [23, 30]. The database also contains data that are (i) crowdsourced using the FoodSwitch smartphone application (~ 30%) and (ii) provided directly by the food industry (~ 10%) [29].

As FoodSwitch contains only nutrition information for packaged foods that carry a nutrition information panel, we extracted energy content information for unpackaged, unbarcoded products reported by households (i.e. scanning guide items) from similar food or beverage subcategories in Australian Food and Nutrient database (AUSNUT) 2011–2013 [31]. AUSNUT is a food nutrient database containing nutrient values for 5740 generic foods and beverages with reported consumption in the 2011–2013 Australian Health Survey [31]. Where multiple relevant products were available in the AUSNUT dataset, we used the average energy content of all relevant products.

All products were assigned to food categories based on the categorisation system developed by the Global Food Monitoring Group, which classifies all products into a hierarchical category tree to allow for comparison of nutritionally similar foods [28]. This system classifies each product into a food group (e.g., bread and bakery), category (e.g., bread), subcategory (e.g., flat bread) and minor category (e.g., regular wraps).

### Merging NielsenIQ and FoodSwitch datasets

The first step in merging the two datasets was to exclude products not relevant for the analyses. This included the removal of non-food and beverage products from the NielsenIQ Homescan database, such as medicinal items and cleaning products. We also excluded alcoholic beverages, vitamins and supplements from both databases.

The remaining food and beverage products in the NielsenIQ dataset were then linked with their corresponding nutrient information from FoodSwitch to obtain the energy content and NOVA classification. Initial matching of NielsenIQ Homescan to FoodSwitch was carried out using the unique barcode associated with each product followed by additional steps to further improve the coverage of products purchased by households [23, 32]. This included linking products by product name only, then by product name after removal of irrelevant descriptors (e.g., shape and size information). For unpackaged foods and beverages (i.e., those without a barcode), the energy content was matched to information from AUSNUT. After these additional steps were applied, the match rate across the NielsenIQ and FoodSwitch datasets was approximately 96.6% according to the total volume of products purchased over the five-year period. There was similar coverage across each of the five years (2015 = 96.1%, 2016 = 96.6%, 2017 = 96.8%, 2018 = 97.0% and 2019 = 96.6%).

### Level of processing classification

The NOVA system categorises products into four categories based on the extent and purpose of industrial food processing. These include Group 1: Unprocessed or minimally processed foods (e.g., rice, meat, fish, milk, eggs, fruit, vegetables, nuts, and seeds); Group 2: Processed culinary ingredients (e.g., sugar, oils, butter); Group 3: Processed foods (e.g., canned fruit, canned fish, freshly baked bread, some cheeses); and Group 4: Ultra-processed foods (e.g., mass produced packaged breads, cookies/pastries, confectionery, savoury snacks, reconstituted meat products and sugar sweetened beverages) [3].

We categorised products matched across the NielsenIQ and FoodSwitch datasets (96.6% of all product units) into two groups based on level of processing: (1) ultra-processed (NOVA Group 4) and (2) non-ultra-processed (NOVA Group 1 to 3). Using previously described methods, products with ingredient list information (~ 93% of all product units) were classified as ultra-processed if they contained ultra-processed ingredients i.e. ingredients that are never or rarely used in household kitchens or additives that function to make foods more palatable and/or appealing, including flavours, emulsifiers, modified starches, vegetable gums, stabilisers and artificial sweeteners [7, 33, 34]. A full list of ingredients used to identify ultra-processed foods is provided in Supplementary Table 1. For products missing ingredient list information (~ 3% of all product units), we applied the NOVA system using food category information [3]. For example, any eggs, legumes, herbs, unprocessed and unflavoured meat, poultry and seafood products were categorised under Group 1: unprocessed or minimally processed foods, whereas sugar sweetened beverages, sweet and savoury snack foods, chocolate, ice-cream, breakfast cereals were categorised under Group 4: Ultra-processed foods.

### Socio-economic status

The SES of participating households was assessed based on their postcode using the Index of Relative Social Advantage and Disadvantage (IRSAD), which is a Socioeconomic Index for Areas (SEIFA) as defined by the Australian Bureau of Statistics (ABS) [35]. IRSAD ranks geographic areas according to relative socio-economic advantage and disadvantage using a range of indicators including education, income, occupation and housing [35]. Using this index, households were divided into quintiles according to SES (Quintile 1: lowest SES; quintile 5: highest SES).

### Statistical analysis

We assessed Australian household purchases of ultra-processed foods in 2019 using two outcome measures, 1) mean per capita purchases (grams/day); the amount of ultra-processed foods purchased daily per person and 2) contribution to total daily energy purchases (% energy); amount of energy purchased from ultra-processed foods as a proportion of total energy purchased from all grocery purchases. The major food categories contributing to total purchases of ultra-processed foods across Australian households were identified and ranked according to their relative contribution (%) to total daily energy purchases. We also explored differences in household purchases of ultra-processed foods across quintiles of SES. Differences in mean per capita purchases of ultra-processed foods across each of the SES quintiles were assessed using survey-weighted linear regression.

We also assessed changes in purchases of ultra-processed foods by SES between 2015 and 2019. Weighted linear mixed models were fit with the household as a random effect nested within the region with the year as a fixed effect. Purchases of ultra-processed foods and the contribution of ultra-processed foods to total purchases of energy were treated as dependent variables in the models. The model included the number of children in the household, the number of adults in the household, and life stage of the household (young singles & couples, young families, mixed families, older families, older singles & couples, adult households) as a fixed effect and were adjusted for in the model as these factors are likely to impact purchasing behaviours. To examine differences over time between SES groups, weighted linear mixed models were fit with an interaction term between SES and year. Using the parameters from the models, predicted means using a household of two adults and one child in the purchases of ultra-processed foods (g/d per capita) and contribution of ultra-processed food to total purchases of energy (% energy) among households of different socio-economic status over time were calculated. Survey weights (provided by Nielsen IQ) were applied throughout all analyses to ensure annual purchases were representative of the SES, demographic, and geographic composition of the Australian population [23].

All statistical analyses were performed using R Studio (version 1.4.1106) & R (version 4.1.0). Packages *survey* and *lme4* were used for this analysis. A two-sided *p*-value of < 0.05 was considered statistically significant.

## Results

### Household characteristics

In 2019, the mean (SD) household size was 2.6 (1.4) persons. The most common family composition was older singles and couples (all adults > 45 years) (45% of all households) and approximately 69% of households resided in metropolitan areas. Household characteristics in 2019 were very similar to the socio-and-demographic characteristics of the Australian population (data not shown) [23, 25]. The characteristics of households were largely constant over the five-year period and are presented in Supplementary Table 2.

### Amount of ultra-processed foods purchased by Australian households in 2019

In 2019, the per capita mean ± SD total grocery purchases acquired and brought home by Australian households was 881.1 ± 511.9 g/d, equivalent to 5114 kJ ± 2620 kJ/day. Of this, 424.2 ± 319.0 g/d was attributable to purchases of ultra-processed foods, which accounted for 56.4% of total energy purchased. The top 10 food categories containing ultra-processed foods together contributed to 39.9% of total energy purchases (Table 1). On average, the largest food category contributors to ultra-processed foods included mass-produced, packaged breads (40.2 g/d per capita, 8.2% of total energy purchases), chocolate and sweets (15.8 g/d per capita, 5.7%), biscuits and crackers (14.9 g/d per capita, 5.7%), ice-cream and edible ices (26.3 g/d per capita, 4.3%), breakfast cereals (12.1 g/d per capita, 3.8%), and processed meats (21.3 g/d per capita, 3.8%) (Table 1).

**Table 1:** Major food categories contributing to purchases of ultra-processed foods across Australian households in 2019

**Food category rank**^a^	**Food category**	**Total weight of products purchased** **(g/d per capita)** **Mean (95% CI)**	**Contribution to total energy purchased** **(% energy)** **Mean (95% CI)**
1	Mass-produced, packaged breads	40.2 (39.1, 41.3)	8.2 (8.0, 8.3)
2	Chocolate and sweets	15.8 (15.3, 16.2)	5.7 (5.6, 5.8)
3	Biscuits and crackers	14.9 (14.5, 15.2)	5.7 (5.6, 5.8)
4	Ice cream and edible ices	26.3 (25.4, 27.2)	4.3 (4.2, 4.4)
5	Breakfast cereals	12.1 (11.7, 12.5)	3.8 (3.7, 3.9)
6	Processed meats	21.3 (20.7, 21.8)	3.8 (3.7, 3.9)
7	Cakes, muffins and pastries	10.9 (10.6, 11.2)	2.9 (2.9, 3.0)
8	Margarines and spreads	5.1 (4.9, 5.3)	2.1 (2.1, 2.2)
9	Potato-based snacks and chips/crisps	4.0 (3.9, 4.1)	1.7 (1.7, 1.8)
10	Soft drinks	110.0 (104.3, 115.7)	1.7 (1.6, 1.8)
Other		183.4 (179.7, 187.1)	18.9 (18.7, 19.1)

^a^Rank = Food categories are ranked in order of their contribution to the total energy purchased in 2019 (%), from highest to lowest. Results for the top 10 food categories are shown separately, with the remaining 75 food categories summed together to simplify data presentation

### Purchases of ultra-processed food according to SES in 2019

**Fig. 1:**
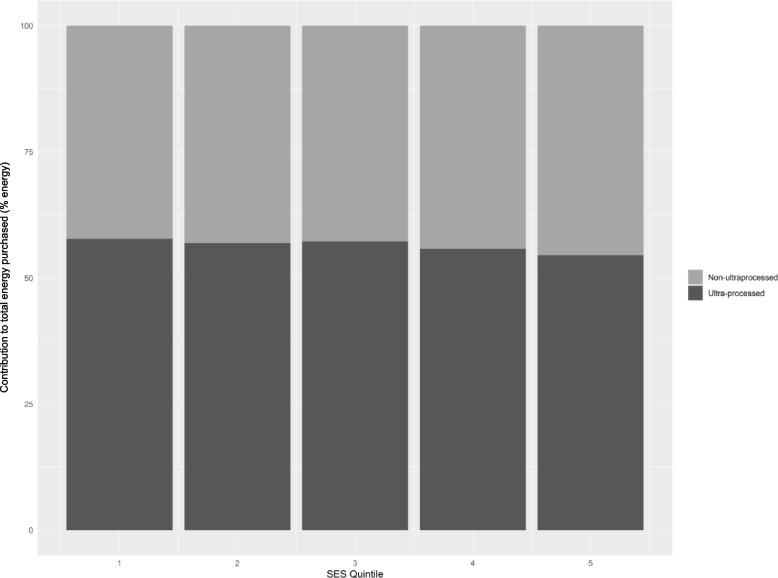
Mean contribution of ultra-processed foods to total daily energy purchased by SES (% energy). The dark grey area represents total proportion of daily energy purchases (%) from ultra-processed foods and beverages. SES; socio-economic status – Quintile 1: lowest socio-economic status, Quintile 5: highest socio-economic status

In 2019, purchases of ultra-processed foods followed an inverse gradient with household SES, such that households in the lowest SES quintile purchased the most ultra-processed foods at 495.0 g/d per capita (95%CI, 475.3—514.3 g/d, 57.7% of total energy purchases), and households in the highest SES quintile purchased the least at 354.0 g/d per capita (338.3—369.9 g/d, 54.5%) (Fig. 1). Per capita purchases of ultra-processed foods were significantly higher for the lowest SES households compared to all other SES quintiles (*P* < 0.001); and the most pronounced difference was between households of the lowest and highest SES, with a mean difference of 140.7 g/d per capita (95%CI, 115.6 – 165.8 g/d) and 3.3% (95%CI, 2.2%—4.4%) in total purchased amounts and proportion of energy purchased, respectively.

### Change in purchases of ultra-processing foods by SES, 2015 to 2019

**Fig. 2:**
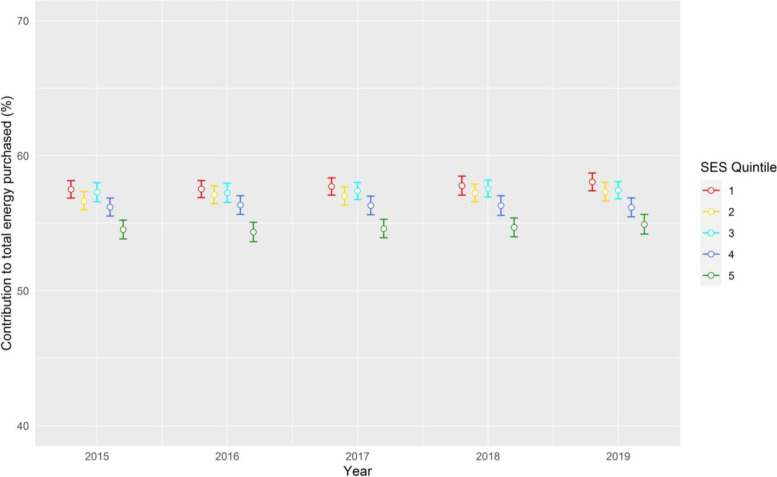
Change in contribution of ultra-processed foods to total daily energy purchased (% energy) between 2015 and 2019, by socio-economic status. SES; socio-economic status – Quintile 1: lowest socio-economic status, Quintile 5: highest socio-economic status

Across the five-year period, purchase data was collected from a total of 10,008 individual households. In each year, there is a similar pattern of lower SES households purchasing the highest amounts of ultra-processed foods and highest proportion of ultra-processed foods as a proportion of total daily energy purchased (Fig. 2). Across all households, there was a very small although statistically significant trend toward a greater contribution of ultra-processed to total energy purchases over time, increasing by 0.1% **(**95% CI, 0.0 – 0.1%, *P* < 0.001) per year between 2015 to 2019, however, these annual changes were not significant for total purchases (g/d per capita) of ultra-processed foods. Similarly, there were no significant differences in trends of ultra-processed food purchases over time between households of different SES, with differences between SES groups remaining fairly stable over this period.

## Discussion

Using large and nationally representative consumer and food supply datasets, this study quantified the amount and main types of ultra-processed foods purchased by Australian households over the past five years and their contribution to apparent energy intake. We found that ultra-processed foods accounted for ~ 55% of total energy from all foods and beverages purchased from retail outlets. This was largely driven by purchases of mass-produced packaged breads, chocolate and sweets, biscuits and crackers, and ice-cream and edible ices. We found a significant socio-economic gradient in the purchases of ultra-processed foods, with the lowest SES households purchasing the most.

A key finding from our analyses is that ultra-processed foods accounted for the majority of Australian household grocery purchases, and that purchases have not reduced in recent years. This is a concern, particularly considering growing evidence links ultra-processed food consumption to non-communicable diseases including heart disease, type 2 diabetes and some types of cancer [5, 13, 36]. Our finding that ultra-processed foods accounts for a large portion of energy in the average Australian diet is relatively consistent with prior research, including a study using nationally representative dietary intake data from 2011–12, which found that 42% of total energy intakes in the average Australian diet is attributable to ultra-processed foods [7]. Our findings also align with prior research conducted in other high-income countries, with ultra-processed foods making up 57% of total energy intake in the US in 2017/2018 [37] and 54% in the UK between 2008—2016 [38]. The large dietary share of ultra-processed foods is likely driven by the fact these products are highly palatable, shelf-stable, convenient and affordable, and are often heavily marketed and promoted by food companies and supermarket retailers [7, 39].

Importantly, this study has shown that there is a wide variation in the types of foods that contribute to ultra-processed food purchases. These range from foods that are already considered by traditional nutrient based food classification systems as unhealthy such as confectionary and sugary drinks, through to staple foods that are generally considered healthy such as mass-produced packaged breads. These findings clearly demonstrate the need for additional investigations to elucidate how the extent of processing affects the health impact of foods *above and beyond* that is conferred by their nutrient profile alone [40]. Answer to this question will have important implications for the design of dietary guidelines and policy responses aimed at enhancing population dietary intakes. In the meantime, given the abundant observational evidence linking intake of ultra-processed foods with worse health outcomes, measures to reduce ultra-processed food intake have already been put in place in some countries. These include limits for the availability of ultra-processed foods in certain institutional setting such as schools and hospitals, restrictions on advertising and levies/taxes for some ultra-processed foods such as sugary drinks [39, 41–43]. Introduction of subsidies for healthy foods that reduce the relative cost of healthy wholefoods such as fruits, vegetables and grains may be an additional policy that could help to reduce intakes of ultra-processed foods through shifting diets towards these healthier alternatives [39, 42].

Another important finding from this research is the association between purchases of ultra-processed food by SES. Consistent with previous research, we found that households from more disadvantaged socio-economic backgrounds were more likely to purchase the highest volumes of ultra-processed foods [41, 44, 45]. While the differences according to proportion of total of energy purchases was relatively small (likely driven by the fact that these households tend to purchase the largest volumes of grocery purchases [23, 24], thereby reducing the relative contribution of ultra-processed foods to total purchases), it is still clear there are differences in household behaviours by SES when it comes to purchases of ultra-processed foods. Given the SEIFA index for SES is based on area-level disadvantage, it is likely that these findings are influenced by a household’s access to healthy and affordable grocery retail outlets [46–48]. These findings highlight the need for upstream policies, such as enhancing the supply chain to ensure widespread availability for healthy foods such as fruit and vegetables, to reduce diet driven health inequalities in Australia [39, 41–43].

A key strength of the study was the use of objective and contemporary food and beverage purchase data from a large, representative sample of Australian households. Moreover, we were able to match this data to a large, up-to-date packaged food database containing product-level data collected from major Australian grocery retailers, who represent about 80% of total market share in Australia [49]. Together, these datasets ensured our results reflected both the purchasing habits of Australian households and the prevalence of ultra-processed foods in the Australian food supply.

A limitation of the analyses is that household purchasing data is only a proxy for consumption. The purchases recorded do not equate to total dietary intake, because we did not assess consumption of foods and beverages purchased and consumed outside of the home, such as from cafés, take-away restaurants and food delivery services. These out-of-home purchases represent a growing area of household food spend, particularly since the COVID-19 pandemic, which saw a dramatic increase in the marketing and subsequent purchases of foods from online food and meal delivery platforms [50, 51]. Given the type of foods and beverages sold across these platforms are largely skewed toward unhealthy products [52], the growth in online food delivery has potentially further increased intakes of ultra-processed foods across the Australian population. Understanding the current amount of ultra-processed foods consumed from food and meal delivery services is an important area for future research to estimate the presence of ultra-processed foods across the whole diet [50, 51]. A further limitation is the likely presence of under-reporting of purchases, which has been previously estimated to be around 10–20% for the NielsenIQ Homescan database [53, 54]. While we sought to minimize this impact by setting threshold criteria for grocery expenditure, some under-reporting may still be present such as from products purchased outside regular grocery shops. However, the magnitude of this potential effect is likely small given under-reporting rates are less than 20% [53, 54].

Moreover, we acknowledge that the current literature reports a number of differing interpretations for how to apply the NOVA classification system, many of which lack explicit and objective definitions and/or classifications [55]. This ambiguity has been shown to impact how accurately and reliably the NOVA classification system is applied [40, 56]. In our study, we identified ultra-processed food products using product-specific ingredient lists and food category information [7, 33, 34], which aligns with the recommended approach that has been used in prior observational studies linking ultra-processed food consumption to health outcomes [7, 34]. However, it is possible we may have not included all scientific names for previously defined ultra-processed ingredients given there is little regulation in Australia for how these ingredients should be named on a product. Moreover, this ingredient-based method could not be applied to unpackaged food products as ingredient list information for these products is not provided. As we were required to rely on food category assumptions about processing for these products, this may have resulted in less accurate processing classifications. However, this is unlikely to have had a substantive impact on the results given these products represented only ~ 3% of all product units and were largely from unprocessed or minimally processed food categories like fresh fruit, vegetables and fresh, unpackaged bakery items. Lastly, given our study analyzed packaged food and beverages available in Australia and purchased by Australian households, our findings may not be generalizable to other countries.

## Conclusion

In this analysis of grocery purchases from a nationally representative sample of Australian households, ultra-processed foods consistently made up the majority of energy purchased over five years from 2015–2019. Purchases of ultra-processed foods were greater for the lowest SES households. The high proportion of ultra-processed foods in Australian diets highlights the need for policy actions that specifically target a reduction in unhealthy ultra-processed foods to help achieve healthier Australian diets and reduce high rates of diet-related NCDs.

## Supplementary Information

**Supplementary Table 1**. List of ingredients found exclusively in ultra-processed products. **Supplementary Table 2.** Household characteristics of the NielsenIQ Homescan Consumer panel between 2015 and 2019.

## Data Availability

The data that support the findings of this study are available from NielsenIQ and FoodSwitch, but restrictions apply to the availability of these data, which were used under license for the current study, and so are not publicly available.

## Abbreviations

SES: Socio-economic status
NCD: Non-communicable disease
IGA: Independent Grocers of Australia
AUSNUT: Australian Food and Nutrient Database
IRSAD: Index of Relative Social Advantage and Disadvantage (IRSAD)
ABS: Australian Bureau of Statistics
