# Supplementary material for: Socio-economic difference in purchases of ultra-processed foods in Australia: an analysis of a nationally representative household grocery purchasing panel
Source: Int J Behav Nutr Phys Act. 2022 Dec 12;19:148. doi: 10.1186/s12966-022-01389-8 (PMC9742014; doi:10.1186/s12966-022-01389-8)
Supplement: Supplementary file 1 — Supplementary Table 1. List of ingredients found exclusively in ultra-processed products. Supplementary Table 2. Household characteristics of the NielsenIQ Homescan Consumer panel between 2015 and 2019. [file 12966_2022_1389_MOESM1_ESM.docx]

**Supplementary Table 1**. List of ingredients found exclusively in ultra-processed products.

| Agar | Glycerol |
| --- | --- |
| Agent  (anti-caking, de-foaming, firming, glazing, leavening, raising) | Gums (guar, vegetable, xanthan) |
| Aspartame | Humectant |
| Binder | Hydrogenated/interesterified oil |
| Beta carotene | Improver |
| Caffeine | Inositol |
| Carbon dioxide | Invert sugar |
| Carrageenan | Lactose |
| Casein | Maltitol |
| Charcoal | Maltose |
| Coffee essence | Mannitol |
| Colour/Color | Menthol |
| Confectioners glaze | Modified (maize/potato) starch |
| Corn syrup (incl. high fructose) | Monoglyceride |
| Dextrose | Oligosaccharides |
| Dextrin | Pectin |
| Disodium guanosinemonophosphate | Propane |
| Emulsifier | Propellant |
| Emulsifying salt | Protein powder/isolate  (including hydrolysed/hydrolyzed) |
| Erythritol | Sodium nitrite |
| Esters | Sorbitol |
| Extract | Soy lecithin |
| Flavour/Flavor | Stabiliser/stabilizer |
| Food additive | Sucralose |
| Fructose | Sucrose |
| Fruit juice concentrate | Sweetener |
| Glucose | Thickener |
| Glucuronolactone | Trehalose |
| Gluten | Triglycerides |
| Glycerine | Xylitol |

**Supplementary Table 2.** Household characteristics of the NielsenIQ Homescan Consumer panel between 2015 and 2019

| **Household characteristics** | **2015** | **2016** | **2017** | **2018** | **2019** |
| --- | --- | --- | --- | --- | --- |
|  | **n = 6,970** | **n = 7,348** | **n = 7,472** | **n = 7,373** | **n = 7,535** |
| Household size, mean (SD) | 2.7 (1.4) | 2.7 (1.4) | 2.7 (1.4) | 2.6 (1.4) | 2.6 (1.4) |
| No of adults (>=18yrs) in household, mean (SD) | 2.1 (1.0) | 2.2 (1.0) | 2.1 (1.0) | 2.1 (1.0) | 2.1 (1.0) |
| No of children (<18yrs) in household, mean (SD) | 0.6 (1.0) | 0.6 (1.0) | 0.5 (0.9) | 0.5 (0.9) | 0.5 (0.9) |
| SEIFA (IRSAD)^1^, n (%) |  |  |  |  |  |
| 1 | 1,439 (21%) | 1,492 (20%) | 1,521 (20%) | 1,503 (20%) | 1,536 (20%) |
| 2 | 1,358 (19%) | 1,481 (20%) | 1,504 (20%) | 1,471 (20%) | 1,502 (20%) |
| 3 | 1,385 (20%) | 1,493 (20%) | 1,504 (20%) | 1,477 (20%) | 1,504 (20%) |
| 4 | 1,430 (21%) | 1,469 (20%) | 1,496 (20%) | 1,497 (20%) | 1,524 (20%) |
| 5 | 1,358 (19%) | 1,413 (19%) | 1,447 (19%) | 1,425 (19%) | 1,469 (19%) |
| Geographical location, n (%) |  |  |  |  |  |
| Metropolitan | 4,807 (69%) | 5,080 (69%) | 5,157 (69%) | 5,085 (69%) | 5,225 (69%) |
| Regional | 2,163 (31%) | 2,268 (31%) | 2,315 (31%) | 2,288 (31%) | 2,310 (31%) |

^1^IRSAD; Index of Relative Advantage and Disadvantage. continuous SEIFA IRSAD scores (0–1000) were categorised into quintiles (1-5), with a higher score indicating higher relative level of socio-economic advantage.
